# Cost–consequence analysis of early vs. delayed natalizumab use in highly active relapsing–remitting multiple sclerosis: a simulation study

**DOI:** 10.1007/s00415-024-12723-4

**Published:** 2025-01-17

**Authors:** Hernan Inojosa, Dirk Schriefer, Nils-Henning Ness, Anja Dillenseger, Katja Akgün, Tjalf Ziemssen

**Affiliations:** 1 Department of Neurology, Center of Clinical Neuroscience, University Hospital Carl Gustav Carus, Technische Universität Dresden, Fetscherstr. 74, 01307 Dresden, Germany; 2 Hexal AG, Holzkirchen, Germany

**Keywords:** Multiple sclerosis, Treatment strategies, Markov model, Cost–consequence, Health economics, Economic evaluation

## Abstract

**Background:**

Natalizumab (NAT) is an established disease-modifying therapy (DMT) for highly active multiple sclerosis (MS). However, its use involves complex decision-making, often leading to initial use of lower efficacy therapies. Recently, the first biosimilar NAT was approved, enabling competitive pricing. This study assessed the societal implications of initiating NAT in various scenarios through a cost–consequence analysis.

**Methods:**

A 10-year Markov model based on the Expanded Disability Status Scale (EDSS) was employed, with 11 health states, annual cycles, and half-cycle correction. The cohort had an initial age of 36 years and 70% females. NAT was compared to common initial therapies (glatiramer acetate, teriflunomide, dimethyl fumarate, and fingolimod). Scenarios included continuous use, early (after 1 year), and delayed (5 years) switch to NAT. Baseline characteristics and probabilities for clinical and economic outcomes were derived from clinical trial data, published literature, and other available sources.

**Results:**

Continuous NAT use resulted in the highest time spent on low EDSS levels, fewer relapses, reduced years of life lost due to disability, and a higher employment rate over a 10-year period. Switching to NAT after 1 year yielded outcomes similar to continuous NAT use. Despite higher DMT costs, disease management costs, including indirect costs and non-DMT direct medical costs, were lower in continuous use and early switch to NAT. Late switching resulted in outcomes most comparable to continuous use of the initial DMT.

**Conclusion:**

Continuous and early switch to NAT resulted in better clinical outcomes and lower societal economic burden compared to delayed NAT initiation, indicating potential long-term cost savings.

**Supplementary Information:**

The online version contains supplementary material available at 10.1007/s00415-024-12723-4.

## Introduction

Multiple sclerosis (MS) is a chronic autoimmune disease of the central nervous system, primarily affecting young adults on the peak of their productive life, and leading to varied neurological symptoms that can significantly affect quality of life and productivity [1, 2]. Several disease-modifying therapies (DMTs) have emerged with positive impacts on the disease course, including reduction in inflammatory activity and disability accumulation, thus improving the quality of life of people with MS (pwMS). Among the DMTs with highest efficacy, natalizumab (NAT) has been widely used in treatment of highly active MS in the last years [3–8].

NAT is a monoclonal antibody that selectively inhibits the adhesion molecule α4-integrin, blocking its interaction with the vascular cell adhesion molecule-1 (VCAM-1) and preventing the migration of inflammatory leukocytes across the blood–brain barrier. This action effectively reduces the inflammatory processes that lead to demyelination and axonal damage in MS. Clinical trials and subsequent studies have consistently demonstrated that NAT significantly reduces relapse rates and radiological markers of disease activity more effectively than several baseline treatments [3, 8].

However, the use of NAT is frequently associated with complex decision-making due to its cost and risk profile, including the risk of progressive multifocal leukoencephalopathy [9]. DMT acquisition costs may influence treatment decision in healthcare systems with limited budgets or strict cost-control measures. In such settings, healthcare institutions and authorities may prioritize initial use of lower cost, often lower efficacy DMTs, escalating to more effective DMTs only in case of persistent disease activity [10]. Consequently, there is an ongoing debate regarding the optimal timing for the highly effective DMTs [11–15]. Evidence suggest that controlling inflammatory activity in early phases of the disease may positively affect on future disease progression [14, 16].

Due to the high costs associated with MS management, including diverse direct and indirect medical expenses, particularly the use of relatively expensive high-efficacy DMTs raises significant societal economic considerations. Economic evaluations, such as cost–consequence analyses, can support decision-making in MS management.

Furthermore, the recent introduction the recent introduction of biosimilars, such as Tyruko^®^, the first biosimilar approved for use in MS and developed as a counterpart to NAT, offers new opportunities to improve accessibility and affordability of high-efficacy treatments [17–20]. Biosimilars are developed after the patent expiration of biologic drugs and provide comparable efficacy, safety, pharmacokinetics, and pharmacodynamics to their reference products. By enabling competitive pricing, biosimilars can help lower overall healthcare costs and expand treatment access, particularly in healthcare systems with limited budgets where high drug costs may restrict patient care [10, 20].

Considering the emergence of the first biosimilar for MS, this study aimed to evaluate the societal clinical and economic implications of initiating NAT in various treatment scenarios. The long-term impact of the timing of NAT, including the potential effects of delaying this DMT due to other, less effective options, was assessed.

## Methods

A discrete-time, multi-state Markov cohort model was developed to simulate clinical and economic outcomes over a 10-year horizon and to compare treatment with NAT against other DMTs using a cost–consequence analysis approach [21–23].

### Model structure

The model structure comprised 11 health states delineated by integer Expanded Disability Status Scores (EDSS) [24]. These included EDSS 0–9 for patients with relapsing–remitting multiple sclerosis (RRMS) for both a natural history and a treatment-adjusted model, as well as one Markov state for death from all causes. The model employed annual cycles, through which transitions between disease states were simulated based on treatment efficacy, disease activity, and progression (Supplementary Figure S1). In the model, patients may either experience a relapse or progression to death from any given health state. The EDSS could increase, decrease, or remain unchanged from one annual cycle to the next. Treatment discontinuation occurs upon reaching an EDSS threshold of 7 or higher, or for other reasons in any health state. After discontinuation of treatment, patients continue with best supportive care, following the natural history of the disease. In the Markov model, clinical and economic data were assigned to each health state, aggregated over time, and evaluated from a societal perspective. To ensure accurate estimation of patient distributions and outcomes over the simulation period, a half-cycle correction was applied to adjust for the timing of transitions. A detailed description of the model structure and its underlying assumptions is available in the supplementary material (Supplementary Table S1, Supplementary Figure S1).

### Intervention and comparators

The primary intervention was intravenous NAT, while the comparators were glatiramer acetate (GA), teriflunomide (TER), dimethyl fumarate (DMF), and fingolimod (FTY). According to their efficacy profiles, GA, TER, and DMF are considered as baseline DMTs, while FTY is used in highly active MS. Treatment regimens were simulated in three scenarios: (i) a continuous treatment with each DMT (without treatment switching), (ii) an early switch/ escalation to NAT (after one year of initial treatment), and (iii) a late switch/escalation to NAT (after 5 years of initial treatment). Standard approved treatment doses were employed in all simulations.

The selection of DMF, GA, TF, and FTY was based on their status as the most frequently administered first-line DMTs in the 12- and 24-month periods preceding NAT initiation in Germany, according to pharmacy dispensing data and personal communication [15]. The sequence and timing of 1-year and 5-year delayed initiation of NAT were informed by the operationalization and results of a recent observational cohort study regarding the timing of high-efficacy DMTs and comparable prior economic evaluations [14, 25]. A 10-year time horizon was chosen supported by previous health economic models, available data, and clinician input.

### Model parameters (input)

The input parameters utilized in the model are broadly categorized into five distinct groups: (1) baseline population characteristics, (2) natural history transition probabilities, (3) treatment-specific effects, (4) MS-specific disability weights and mortality data, and (5) cost and work productivity data. The modelling approach and selection of input data sources were informed by empirical and expert recommendations, as well as insights gleaned from previous economic evaluation studies (cost–consequence analyses) that employed comparable Markov models [23, 25, 26].

#### Population characteristics

At model entry, the target population had an average age of 36.0 years, a female-to-male ratio of 70:30, an RRMS disease course, and an EDSS range of 0–6, based on data from the AFFIRM study [3]. The detailed percentage distribution across all health states at baseline is given in Supplementary Table S2, corresponding to a mean EDSS of 2.3 ± 1.2 [3].

#### Transition probabilities (natural history)

The annual transition probabilities for movement between EDSS states were derived from the British Columbia Multiple Sclerosis Natural History Database, as reported by Palace and colleagues [27] (Supplementary Table S3). Annualized relapse rates (ARR) for each EDSS state for the natural history of RRMS are summarized in Supplementary Table S4, and ranged from approximately 0.7 (EDSS ≤ 4) to 0.5 (EDSS > 4) [25, 28, 29].

#### Treatment-specific effects (DMT efficacy)

Treatment-specific effects included efficacy input data pertaining to the reduction of relapse rates, the slowing of disability progression, and the probability of DMT discontinuation. The hazard ratios (HRs) for 6-month confirmed disability accumulation (6-CDP) exhibited a range from 0.46 (NAT) to 0.79 (TER). All HRs, annualized relapse rates (ARRs, ranging from 0.31 to 0.67), and annual discontinuation rates (ranging from 8.7 to 11.2%) were extracted from a network meta-analysis as detailed in Supplementary Table S5.

#### MS-specific mortality and disability weights

Mortality was calculated using age- and sex-specific all-cause mortality and life expectancy data from the general population in Germany and adjusted to the MS population using mortality multipliers by EDSS (Supplementary Table S6) [30, 31]. MS-specific disability weights by EDSS were obtained from Cho and colleagues and represent the severity of health loss in a range from 0 (no health loss) to 1.0 (complete health loss) [32]. Disability weight input data are used to determine the number of Years Lived with Disability (YLD), whereas disease-specific mortality data are relevant for the number of Years of Life Lost (YLL) due to premature mortality.

#### Productivity and cost data

Costs associated with resource utilization and productivity loss in each health state were derived from the German results of a multinational cost study by Kobelt et al., stratified by EDSS health states [33–35]. The cost inputs included direct medical costs (healthcare costs: inpatient stays, outpatient stays, consultation costs, examination costs, costs for DMTs, and other medications), direct non-medical costs (services and informal care costs: community and social services, investments and purchases, and informal care), and indirect costs (short-term absence and long-term absence from work, invalidity, and early retirement). The input data regarding loss of productivity further comprised EDSS-specific estimates of the proportion of patients in employment, the proportion of patients working full-time, the proportion receiving invalidity pension, as well as the number of days of receiving informal care (Supplementary Table S7). The annual costs related to relapse management in Germany were obtained from a study by Ness and colleagues [36].

Annual costs associated with acquiring DMT were based on the German pharmacy price schedules, considering both originator products and follow-on medications such as generics and biosimilars [37]. Prices were weighted by sales volume based on the price data from the LAUER-TAXE^®^ and market shares according to IQVIA PharmaScope^®^ retail sell-out national July 2024. Detailed information on the annual costs for each DMT (ranging from 10,807€ for Teriflunomide to 25,985€ for biosimilar NAT) and the calculation methodology are provided in Supplementary Appendix S8.

All unit costs were adjusted to the 2024 price level using the Consumer Price Index for health care, and future costs were discounted at an annual rate of 3% in accordance with German guidelines [38, 39].

### Clinical and economic outcomes (output)

Clinically, disability-adjusted life years (DALYs) served as outcome measure for the overall disease burden, combining YLL and YLD. Disability was assessed using outcomes based on the EDSS, including the distribution of EDSS scores at 10 years, the percentage of patient time spent in each EDSS level, the proportion of pwMS progressing to an EDSS score greater than seven, and the mean EDSS scores over time. EDSS scores were classified into four groups for the purpose of presenting results: mild disability (EDSS 0–3), requiring a walking aid (EDSS 4–6), wheelchair dependence (EDSS 7), and bedridden status (EDSS 8–9). The average number of relapses per patient served as an additional clinical outcome measure.

Economically, the mean annual costs per patient was reported from a societal perspective. Cost outcomes were segmented into predefined cost (sub)categories and presented as follows: Total costs (sum of all direct and indirect costs), DMT costs, disease management costs (total costs minus DMT costs), direct medical costs, direct non-medical costs, and indirect costs. Costs attributable to relapse events were reported separately. As a productivity outcome measure, the proportion of patients employed (remaining in the workforce) was reported. Microsoft Excel (Microsoft Corporation, Redmond, WA, USA) was employed to develop the Markov model and analyze the data. All graphical representations (bar charts, line graphs, and tornado plots) were generated using GraphPad Prism version 5 (GraphPad Software Inc., La Jolla, CA, USA).

### Sensitivity analysis

One-way (univariate) deterministic sensitivity analyses were performed in the continuous treatment scenarios to assess the robustness of the model under varying conditions. A series of incremental costs were calculated based on the variation of single input parameters related to demographic characteristics, DMT efficacy, and DMT costs by ± 10%. The specific parameters included in these analyses were age, sex distribution, 6-CDP and ARR hazard ratios, DMT discontinuation probabilities, and DMT-related costs.

## Results

### Clinical outcomes of base and switch scenarios

#### Continuous treatment with natalizumab vs. other DMTs

**Fig. 1:**
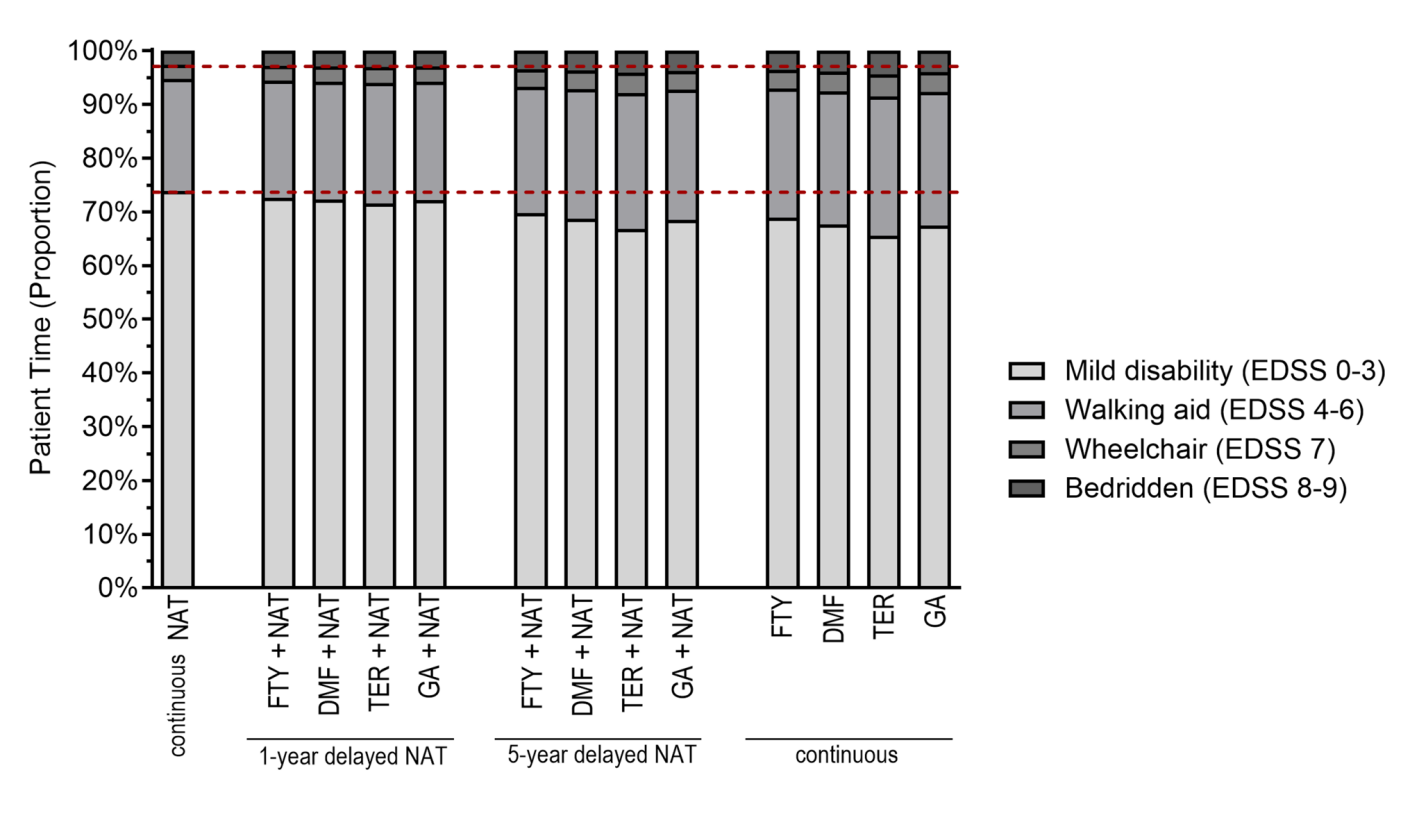
Patient time (in percent) spent in the health states over a 10-year horizon according to DMT and timing of treatment initiation. *NAT* natalizumab, *FTY* fingolimod, *DMF* dimethyl fumarate, *TER* teriflunomide, *GA* glatiramer acetate

**Fig. 2:**
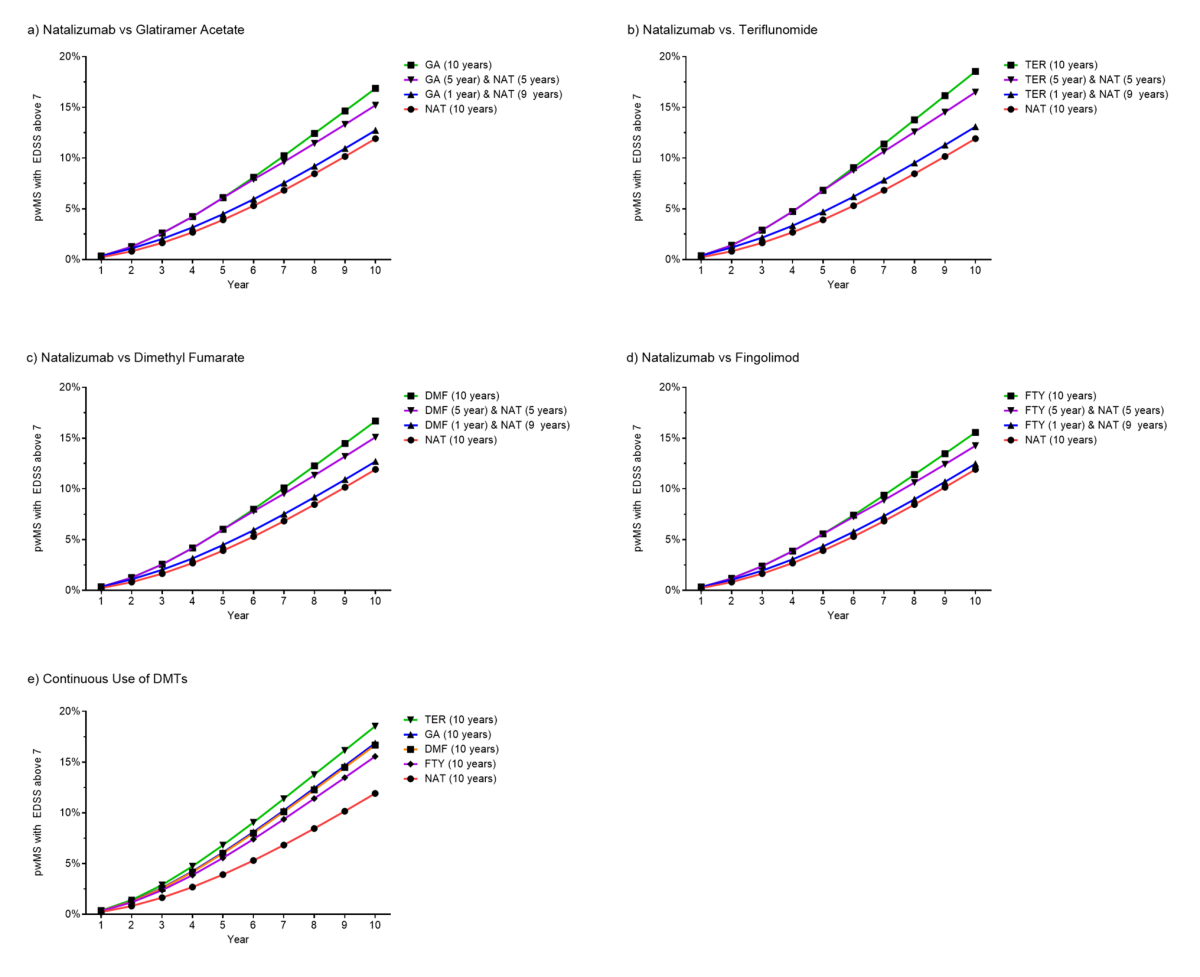
Proportion of people with MS reaching EDSS 7 or higher over a 10-year horizon according to DMT and timing of treatment initiation. *NAT* natalizumab, *GA* glatiramer acetate, *TER* teriflunomide, *DMF* dimethyl fumarate, *FTY* fingolimod

The results of the Markov model indicate that pwMS continuously treated with NAT exhibit improved levels of disability compared to those receiving GA, TER, DMF, or FTY (Table 1). Specifically, pwMS on continuous NAT treatment exhibited lower EDSS scores, with a higher proportion of pwMS experiencing mild disability (EDSS 0–3) at 10 years, and a higher amount of patient time spent in mild levels of disability (Table 1; Fig. 1). The proportion of pwMS experiencing mild disability levels at 10 years was between 7.03 percentage points (pp) and 11.85 pp lower among pwMS receiving continuous treatment with lower efficacy DMTs (GA, TER, DMF, or FTY) in comparison to continuous treatment with NAT. Similarly, the proportion of pwMS requiring a walking aid (EDSS 4–6), a wheelchair (EDSS 7), or becoming bedridden (EDSS 8–9) was consistently lower in the NAT group (Table 1). For instance, after the 10-year treatment period, 11.92% of patients in the NAT group had an EDSS score higher than 7, compared to 15.57%−16.68% in the GA, TER, DMF, and FTY groups (Fig. 2). Moreover, pwMS on NAT experienced fewer relapses, averaging 3.65 relapses over the 10 years, which was lower than the 4.21 relapses observed in the FTY group and notably lower than patients treated with baseline DMTs (4.56–5.27 relapses) (Table 1).

**Table 1:** Clinical outcomes over a 10-year horizon

Clinical outcomes	Reference scenario^a^	Early switch (1/9 years)^b^				Late switch (5/5 years)^c^				Continuous over 10 years^a^			
	NAT	GA/NAT	TER/NAT	DMF/NAT	FTY/NAT	GA/NAT	TER/NAT	DMF/NAT	FTY/NAT	GA	TER	DMF	FTY
Proportion of time spent in health states													
Mild disability (EDSS 0–3)	73.96%	− 1.71 pp	− 2.32 pp	− 1.65 pp	− 1.26 pp	− 5.45 pp	− 7.14 pp	− 5.26 pp	− 4.16 pp	− 6.47 pp	− 8.31 pp	− 6.24 pp	− 5.02 pp
Walking aid (EDSS 4–6)	20.86%	+ 1.16 pp	+ 1.55 pp	+ 1.12 pp	+ 0.87 pp	+ 3.45 pp	+ 4.42 pp	+ 3.34 pp	+ 2.69 pp	+ 4.00 pp	+ 5.01 pp	+ 3.87 pp	+ 3.17 pp
Wheelchair (EDSS 7)	2.56%	+ 0.24 pp	+ 0.34 pp	+ 0.23 pp	+ 0.17 pp	+ 0.91 pp	+ 1.25 pp	+ 0.88 pp	+ 0.67 pp	+ 1.18 pp	+ 1.58 pp	+ 1.14 pp	+ 0.88 pp
Bedridden (EDSS 8–9)	2.62%	+ 0.31 pp	+ 0.43 pp	+ 0.30 pp	+ 0.22 pp	+ 1.09 pp	+ 1.47 pp	+ 1.04 pp	+ 0.80 pp	+ 1.29 pp	+ 1.73 pp	+ 1.24 pp	+ 0.97 pp
Health state distribution after 10 years													
Mild disability (EDSS 0–3)	62.02%	− 1.38 pp	− 1.99 pp	− 1.33 pp	− 0.94 pp	− 5.87 pp	− 8.01 pp	− 5.67 pp	− 4.23 pp	− 9.21 pp	− 11.85 pp	− 8.90 pp	− 7.03 pp
Walking aid (EDSS 4–6)	26.06%	+ 0.57 pp	+ 0.81 pp	+ 0.55 pp	+ 0.39 pp	+ 2.59 pp	+ 3.43 pp	+ 2.51 pp	+ 1.91 pp	+ 4.26 pp	+ 5.24 pp	+ 4.14 pp	+ 3.38 pp
Wheelchair (EDSS 7)	5.25%	+ 0.27 pp	+ 0.40 pp	+ 0.26 pp	+ 0.18 pp	+ 1.14 pp	+ 1.62 pp	+ 1.10 pp	+ 0.78 pp	+ 1.99 pp	+ 2.65 pp	+ 1.91 pp	+ 1.46 pp
Bedridden (EDSS 8–9)	6.67%	+ 0.54 pp	+ 0.77 pp	+ 0.52 pp	+ 0.37 pp	+ 2.14 pp	+ 2.95 pp	+ 2.06 pp	+ 1.54 pp	+ 2.96 pp	+ 3.96 pp	+ 2.84 pp	+ 2.19 pp
Relapses													
Total number	3.65	+ 0.23	+ 0.30	+ 0.15	+ 0.08	+ 0.92	+ 1.13	+ 0.60	+ 0.35	+ 1.39	+ 1.62	+ 0.91	+ 0.56
Mild severity	1.38	+ 0.09	+ 0.11	+ 0.06	+ 0.03	+ 0.35	+ 0.43	+ 0.23	+ 0.13	+ 0.52	+ 0.61	+ 0.34	+ 0.21
Moderate severity	1.76	+ 0.11	+ 0.14	+ 0.07	+ 0.04	+ 0.44	+ 0.55	+ 0.29	+ 0.17	+ 0.67	+ 0.78	+ 0.44	+ 0.27
Severe severity	0.51	+ 0.03	+ 0.04	+ 0.02	+ 0.01	+ 0.13	+ 0.16	+ 0.09	+ 0.05	+ 0.20	+ 0.23	+ 0.13	+ 0.08
Overall disease burden													
DALY	1.82	+ 0.08	+ 0.11	+ 0.08	+ 0.06	+ 0.27	+ 0.36	+ 0.26	+ 0.21	+ 0.33	+ 0.43	+ 0.32	+ 0.25
YLD	1.16	+ 0.07	+ 0.10	+ 0.07	+ 0.05	+ 0.24	+ 0.31	+ 0.23	+ 0.18	+ 0.28	+ 0.37	+ 0.27	+ 0.22
YLL	0.66	+ 0.01	+ 0.01	+ 0.01	+ 0.01	+ 0.04	+ 0.05	+ 0.04	+ 0.03	+ 0.04	+ 0.06	+ 0.04	+ 0.03

Results of different clinical scenarios are represented as absolute differences (including percentage points) compared to 10 years continuous therapy with NAT, as applicable

*NAT* natalizumab, *DMF* dimethyl fumarate, *GA* glatiramer acetate, *TER* teriflunomide, *FTY* fingolimod, *EDSS* Expanded Disability Status Scale, *DALYs* disability-adjusted life years, *YLD* years lived with disability, *YLL* years of life lost, *pp* percentage points

^a^Continuous treatment with selected DMT over 10 years (without treatment switching)

^b^1 year of therapy with initial DMT followed by 9 years with NAT

^c^5 years of therapy with initial DMT followed by 5 years with NAT

Additionally, pwMS treated continuously with NAT lost fewer years of life due to disability, as indicated by lower DALYS (Table 1). This reduction was mainly driven by fewer YLD, while YLL were relatively similar across groups.

**Fig. 3:**
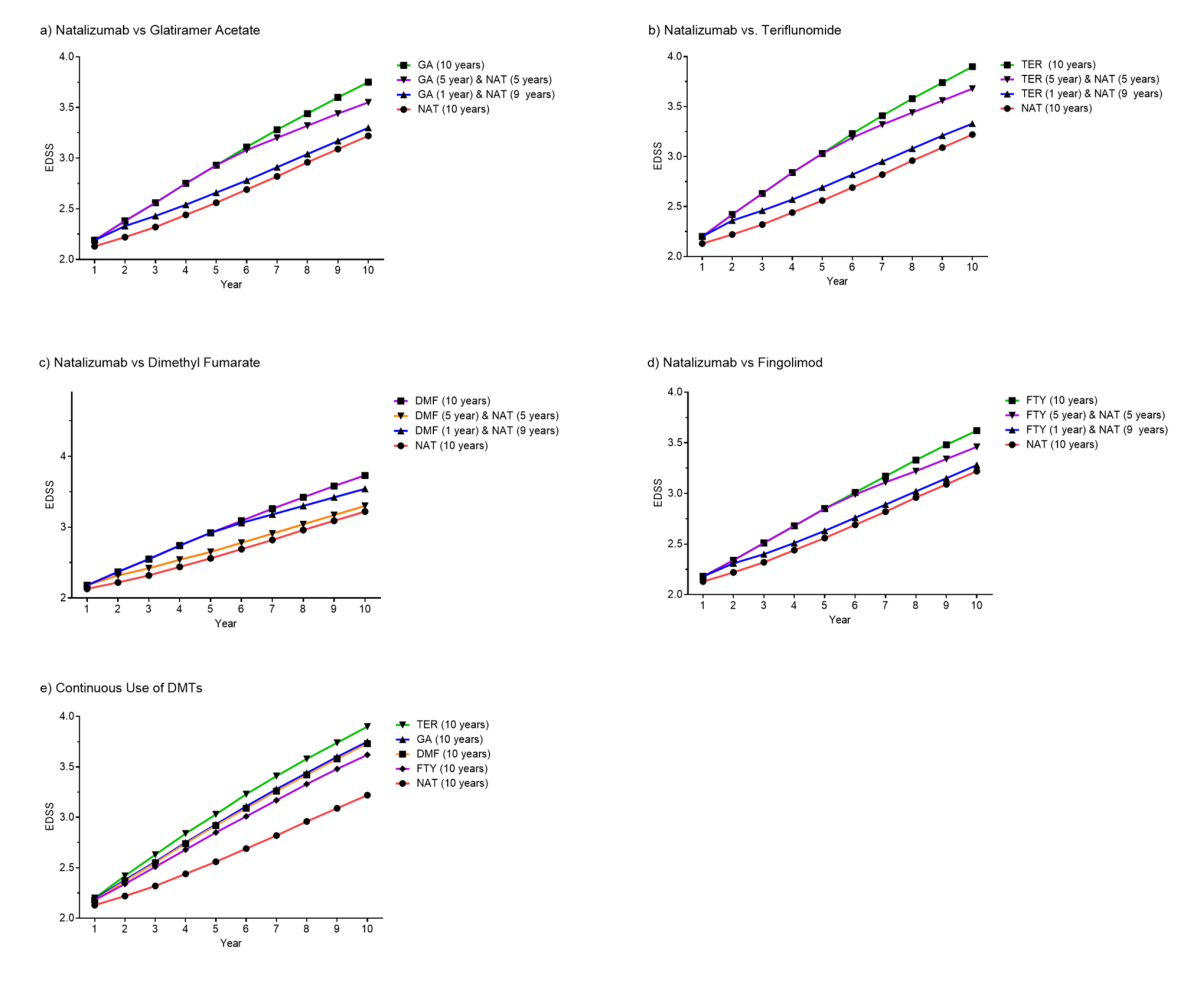
Average EDSS over a 10-year horizon according to DMT and timing of treatment initiation. *NAT* natalizumab, *GA* glatiramer acetate, *TER* teriflunomide, *DMF* dimethyl fumarate, *FTY* fingolimod

The average EDSS scores for pwMS treated continuously with NAT were lower throughout the simulation period and at 10 years, as illustrated in Fig. 3. Specifically, at 10 years, the mean EDSS score for pwMS on continuous NAT treatment was 3.22, compared to 3.62 and 3.90 for the other treatment groups (Fig. 3e).

#### Immediate vs. delayed natalizumab treatment

When considering treatment approaches involving escalation therapies, an early switch to NAT from other DMTs in the study resulted in similar disability and relapse outcomes compared to continuous treatment with NAT, with only slightly worse clinical outcomes in early switchers (Table 1). In late switch scenarios, pwMS exhibited values comparable to continuous treatment with the initial DMTs. Compared to continuous NAT treatment and early switching to NAT, late switchers spent less time at low disability levels and a lower proportion of pwMS experienced mild disability at 10 years (Fig. 1; Table 1). Exemplarily, while the percentage of patient time spent in mild disability levels (EDSS 0–3) was reduced by 1.26–2.32% in early switchers, late switchers showed a reduction of up to 7.14%. Similarly, late switchers had more relapses and a lower proportion of late switchers maintained EDSS scores below 7 (Fig. 2; Table 1). DALYs were also similar to continuous NAT treatment in early switch scenarios, while pwMS in late switch scenarios presented with both more YLD and more YLL (Table 1).

Similarly, early switch to NAT resulted in lower average EDSS scores compared to late switchers (Fig. 3a–d). EDSS values over time and after 10 years were comparable between continuous treatment with NAT and early switchers with only subtle differences.

### Economic outcomes of base and switch scenarios

**Fig. 4:**
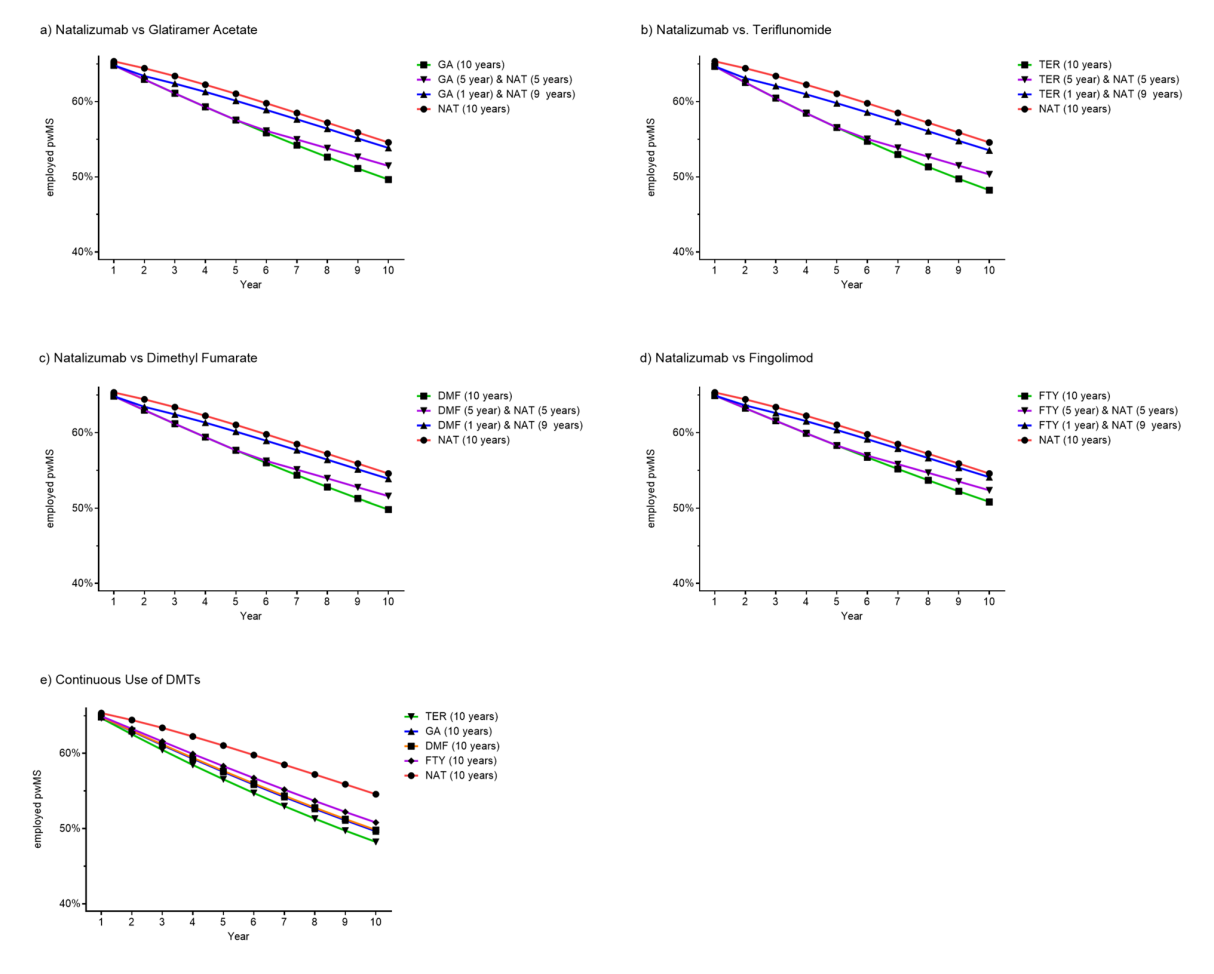
Proportion of employed pwMS over a 10-year period according to DMT and timing of treatment initiation. *NAT* Natalizumab, *GA* glatiramer acetate, *TER* teriflunomide, *DMF* dimethyl fumarate, *FTY* fingolimod

When examining the economic outcomes, the highest proportion of pwMS in employment at 10 years was observed among pwMS continuously treated with NAT (Fig. 4). Those with an early switch (after 1 year) to NAT were also more frequently employed than those who switched later (after 5 years of initial treatment).

**Fig. 5:**
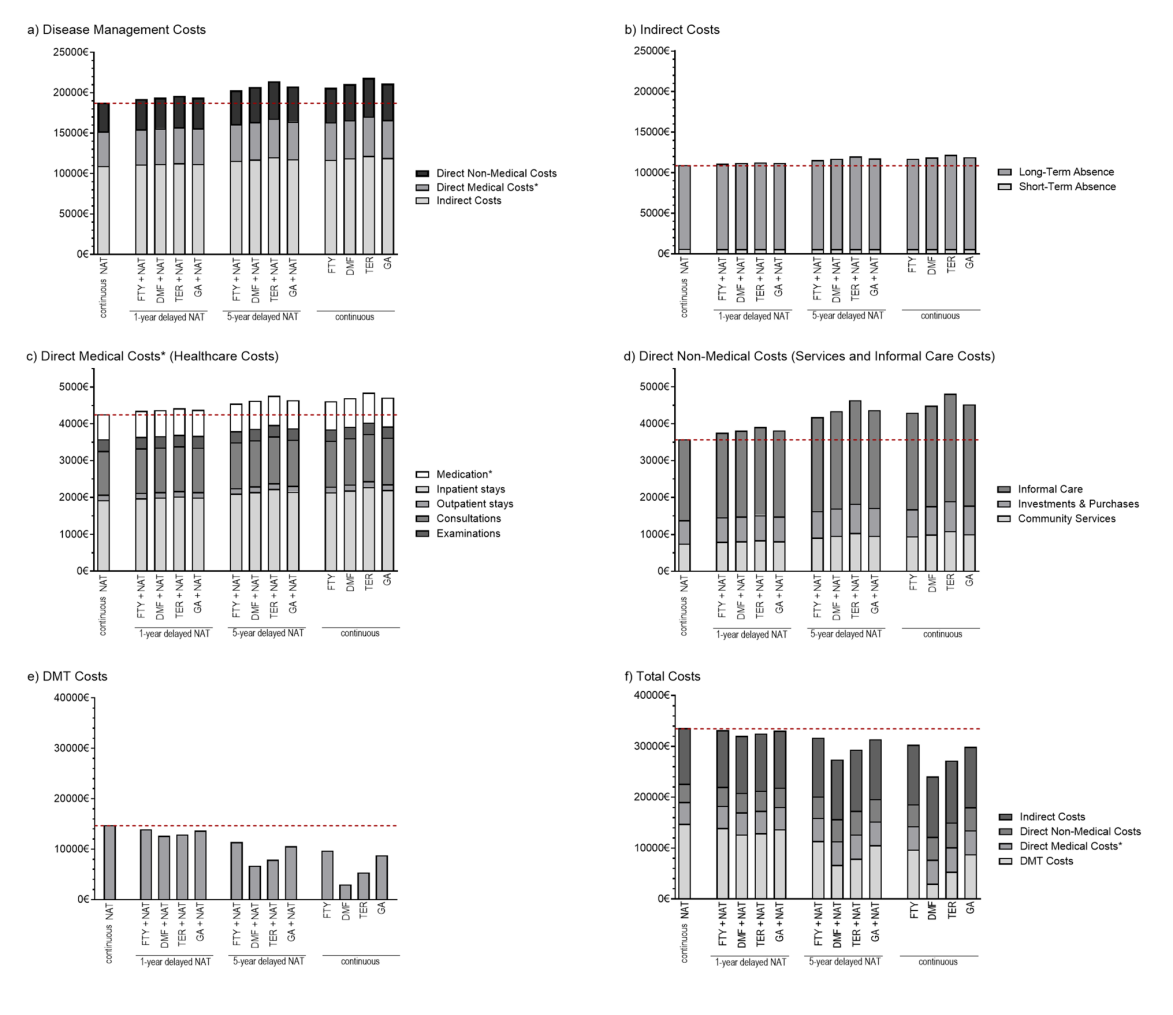
Cost outcomes after a 10-year period according to DMT and timing of treatment initiation. Relapse costs are presented separately in Fig. 6. *Direct medical costs and medication costs excluding DMT-related expenses. Associated DMT-related costs are presented separately in (**e**) and incorporated into the calculation of total costs (**f**). *NAT* natalizumab, *FTY* fingolimod, *DMF* dimethyl fumarate, *TER* teriflunomide, *GA* glatiramer acetate

Moreover, it was observed that annual total costs averaged 30,597€ per pwMS across the different DMTs and scenarios. These costs were higher for groups receiving prolonged NAT treatment or FTY (Table 2; Fig. 5), primarily attributable due to the higher DMT-related costs associated with these treatments (Fig. 5e–f).

**Table 2:** Economic outcomes over a 10-year horizon

Economic outcomes (per pwMS per year in Euros)	Reference scenario^a^	Early switch (1/9 years)^b^				Late switch (5/5 years)^c^				Continuous over 10 years^a^			
	NAT	GA/NAT	TER/NAT	DMF/NAT	FTY/NAT	GA/NAT	TER/NAT	DMF/NAT	FTY/NAT	GA	TER	DMF	FTY
Aggregated annual costs													
Total costs^d^	33,581	98.5%	96.9%	95.5%	98.8%	93.5%	87.4%	81.6%	94.5%	89.2%	81.0%	71.8%	90.3%
DMT costs^e^	14,801	92.5%	87.3%	85.7%	94.2%	71.6%	53.6%	45.2%	77.1%	59.3%	36.2%	20.4%	65.6%
Disease management costs^f^	18,781	103.3%	104.5%	103.2%	102.4%	110.7%	114.0%	110.3%	108.1%	112.7%	116.4%	112.2%	109.8%
Direct medical costs (non- DMT)^g^ (healthcare costs)	4,263	102.8%	103.8%	102.7%	102.0%	108.9%	111.8%	108.6%	106.8%	110.6%	113.7%	110.2%	108.2%
Direct non-medical costs^h^ (services and informal care costs)	3,581	106.7%	109.2%	106.5%	104.9%	122.2%	129.5%	121.4%	116.7%	126.4%	134.5%	125.4%	120.1%
Indirect costs^i^	10,937	102.4%	103.2%	102.3%	101.8%	107.6%	109.9%	107.3%	105.8%	109.0%	111.5%	108.7%	107.0%
Relapse costs^j^	912	7.5%	12.7%	14.3%	5.8%	28.4%	46.4%	54.8%	22.9%	40.7%	63.8%	79.6%	34.4%
Detailed cost distributions													
Inpatient care	1924	103.7%	105.1%	103.6%	102.7%	112.0%	115.8%	111.6%	109.1%	114.3%	118.4%	113.7%	111.0%
Outpatient stays	149	101.3%	101.7%	101.2%	100.9%	104.2%	105.5%	104.1%	103.3%	105.1%	106.4%	104.9%	104.0%
Consultations	1193	101.6%	102.2%	101.5%	101.2%	105.2%	106.8%	105.0%	104.0%	106.2%	107.9%	106.0%	104.8%
Examinations	311	99.8%	99.7%	99.8%	99.8%	99.3%	99.1%	99.4%	99.5%	99.2%	98.9%	99.3%	99.4%
Medication	685	103.9%	105.2%	103.7%	102.9%	112.3%	116.1%	111.8%	109.4%	114.5%	118.7%	114.0%	111.3%
Community services	756	108.3%	111.4%	108.0%	106.0%	128.1%	137.7%	127.0%	121.0%	133.4%	144.1%	132.1%	125.3%
Investments and purchases	627	105.8%	108.0%	105.6%	104.3%	118.9%	124.9%	118.2%	114.3%	122.4%	129.1%	121.6%	117.2%
Informal care	2197	106.4%	108.8%	106.2%	104.7%	121.1%	128.0%	120.3%	115.9%	125.1%	132.8%	124.2%	119.2%
Short-term absence	608	99.1%	98.7%	99.1%	99.3%	97.3%	96.3%	97.4%	98.0%	96.9%	95.8%	97.0%	97.7%
Long-term absence	10,329	102.6%	103.5%	102.5%	101.9%	108.2%	110.7%	107.9%	106.3%	109.7%	112.4%	109.3%	107.5%

Results of different economic scenarios are represented as percentage differences relative to 10 years continuous therapy with NAT

*NAT* natalizumab, *DMF* dimethyl fumarate, *GA* glatiramer acetate, *TER* teriflunomide, *FTY* fingolimod, *DMT* disease-modifying therapy

^a^Continuous treatment with initial DMT (without treatment switching)

^b^1 year of therapy with initial DMT followed by 9 years of treatment with NAT

^c^5 years of therapy with initial DMT followed by 5 years of treatment with NAT

^d^Total costs = disease management costs + DMT costs

^e^DMT costs = DMT acquisition costs

^f^Disease management costs = direct medical costs + direct non-medical costs + indirect costs

^g^Direct medical costs = inpatient care costs + outpatient costs + consultation costs + examination costs + medication costs

^h^Direct non-medical costs = community services + investments + informal care costs

^i^Indirect costs = short-term absence + long-term absence, invalidity and early retirement costs

^j^Relapse costs = costs for managing relapses. It is important to acknowledge that relapse costs are not included in the aggregated cost categories. This is because their incorporation may result in an overestimation of costs (and subsequently of potential benefits), given that relapse-related costs are already partially captured in the referenced data sources for direct and indirect costs (Supplementary Table S6). However, the extent to which relapse costs are covered in these input data is uncertain [25]

**Fig. 6:**
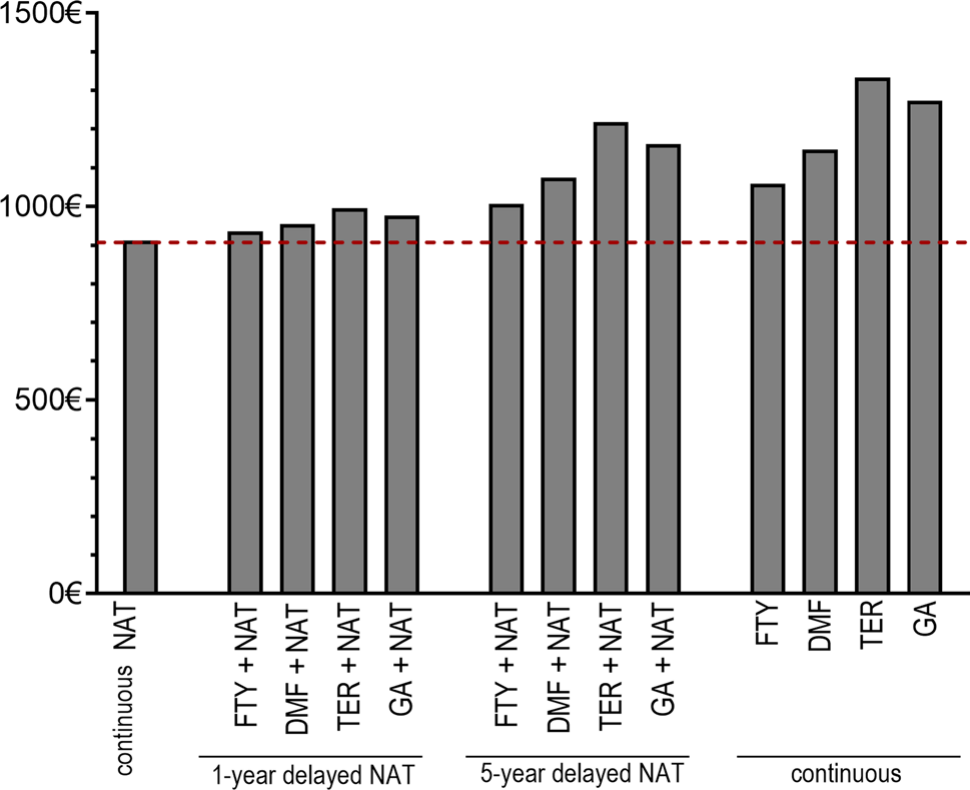
Annual relapse costs after a 10-year period according to DMT and timing of treatment initiation. It is unclear to what extent relapse costs were incorporated into the referenced input data sources utilized for disease management costs. Therefore, to prevent an overestimation of the aggregated costs illustrated in Fig. 5, relapse costs are presented separately. *NAT* natalizumab, *FTY* fingolimod, *DMF* dimethyl fumarate, *TER* teriflunomide, *GA* glatiramer acetate

While the DMT acquisition costs were higher with continuous NAT treatment, the costs associated with disease management were conversely higher for pwMS in the other treatment groups (Fig. 5a). However, direct healthcare costs, cost for services and informal care, and indirect costs were higher in the other treatment scenarios compared with continuous NAT treatment (Fig. 5b–d). Furthermore, an early switch to NAT resulted in economic outcomes that were comparable to those for continuous treatment with NAT. For pwMS in late switch scenarios, economic outcomes were more similar to those of continuous treatment with the initial DMTs (Table 2; Fig. 5).

### Sensitivity analysis

**Fig. 7:**
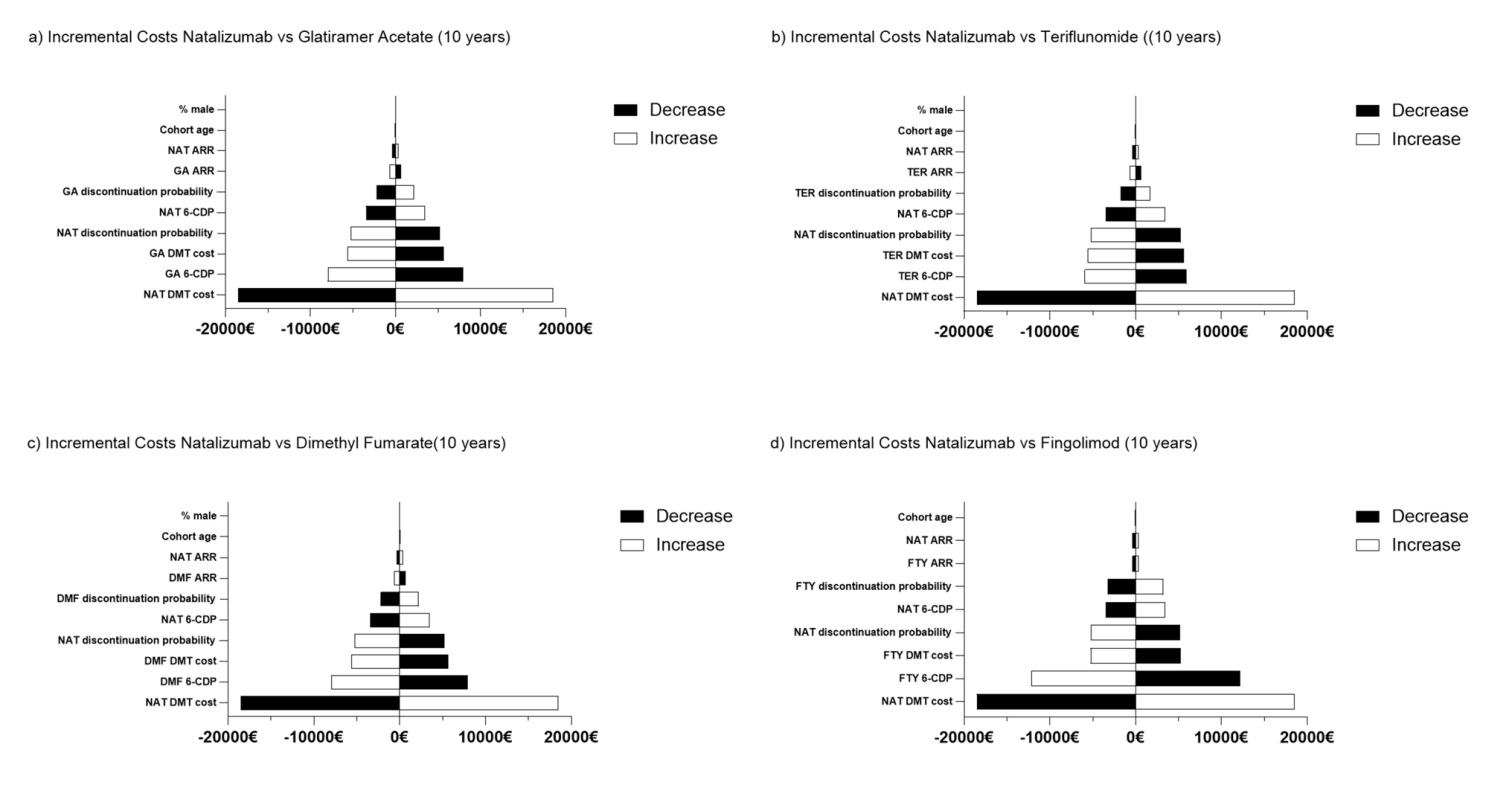
Univariate sensitivity analysis. Incremental costs after increase (white) or decrease (black) in 10% of respective variables are presented for 10 years NAT vs. 10 years of each selected DMT. Costs are presented in 2024 euros. *ARR* annualized relapse ratio, *6-CDP* 6-month confirmed disability progression, *NAT* natalizumab, *GA* glatiramer acetate, *TER* teriflunomide, *DMF* dimethyl fumarate, *FTY* fingolimod

Differences in total costs between NAT and the other DMTs were principally affected by DMT costs (Fig. 7). Alterations in this value yielded constantly the highest incremental costs compared to the other DMTs. Additionally, the outcomes were notably sensitive to the hazard ratios of 6-CDP, DMT costs of comparator DMTs and discontinuation probability of NAT. Changes in annual relapse rates, age or sex distribution, had little-to-no impact on the results.

## Discussion

We demonstrated, through a 10-year Markov model simulation, that continuous treatment with NAT or early switching to NAT resulted in better clinical and economic outcomes compared to continuous treatment with baseline DMTs, FTY or late switching to NAT. While DMT costs were higher in NAT- and FTY-treated pwMS and among early switchers, non-DMT-specific medical costs (healthcare costs), direct non-medical costs (costs for services and informal care), and indirect costs (productivity losses) were lower in these groups.

Our results align with the previous analyses assessing long-term outcomes of early use of highly effective DMTs in real-world settings. The optimal timing of DMT initiation has been widely debated in recent years [40]. In our model, delayed treatment intervals were based on findings from He et al., which demonstrated that early initiation of high-efficacy DMTs was associated with better disability outcomes than delayed initiation [14]. Specifically, their analysis showed that initiating a high-efficacy DMT within 2 years of disease onset resulted in significantly lower EDSS scores after 6 years of treatment compared to delaying treatment for 4–6 years. Further evidence supports the positive effects of early use of DMTs, particularly highly-efficacy DMTs, in reducing inflammatory activity and slowing future disease progression in MS [16, 41–43]. Similarly, a large real-world registry study demonstrated superior long-term outcomes for pwMS who initiated NAT as a first-line DMT compared to those who initially received baseline therapies [44].

We observed low increases in mean EDSS scores after 10 years, corroborating the overall positive impact of early NAT initiation on disease progression evaluated in real-world settings [45–50]. These findings further align with results from post hoc analyses of the AFFIRM trial, which suggested a superiority of NAT after the first year of treatment, and long-term observational effectiveness data [4, 48, 50]. However, our Markov model predicted slightly higher EDSS changes and relapse rates among NAT-treated pwMS compared to findings from a large real-world 10-year follow-up observational program [4]. While this slight discrepancy may reflect inherent limitations of simulation modelling, it may also underscore a conservative nature of our approach, providing cautious estimates that help to avoid overestimating treatment benefits in clinical practice.

Similarly, other simulation-based studies have demonstrated the positive clinical effect of early use of highly effective DMTs, including NAT. Although to our knowledge, no cost–consequence analysis has been published yet, similar results in clinical outcomes were suggested in cost-effectiveness analyses comparing NAT to other DMTs, such as GA, DMF, or FTY [51, 52]. Our results indicate a tendency towards lower disease progression with longer patient times spent in EDSS states of no or mild disability, consistent with most of previous literature comparing NAT to GA, DMF, TER, or FTY over different time horizons. A study by Spelman et al. suggested even more confirmed disability improvements in pwMS treated with NAT compared to FTY over a lifetime horizon [51]. An Italian-based Markov model simulation showed relatively similar proportions of pwMS with lower EDSS values [53]. Moreover, mean EDSS trajectories over time for other DMTs in our model align with the previous Markov simulation models, such as Koeditz et al. who reported similar mean EDSS scores in pwMS treated with DMF or GA over a 10-year period [25].

A recent meta-analysis suggested that NAT and DMF had the highest cost-effectiveness among first-line drugs, and NAT was superior in pwMS with highly active disease [54, 55]. Although our study was designed as a cost–consequence analysis, slightly higher overall costs were observed in continuous or early use of NAT, principally driven by DMT acquisition costs. Previous cost-effectiveness analyses have shown that early escalation to NAT yield lower costs over a lifetime horizon compared to switching among low efficacy therapies [53]. In this analysis, while DMT costs were also higher on pwMS on longer treatment with NAT, however, these were offset by reductions in other and further direct and indirect costs [53]. Another lifetime model demonstrated lower predicted costs for NAT and more QALYs compared to FTY [51]. Similarly, a Markov model with 30 years horizon indicated better cost-effectiveness ratios for NAT compared to GA or interferon [52]. Conversely, a shorter time horizon model indicated lower costs for lower efficacy therapies (e.g., GA or interferon) within 2 years, though these were less effective in reducing number of relapses [56]. Overall, previous research has indicated that cost-effectiveness outcomes tend to be more favorable when observation periods are longer.

Negative economic consequences in MS are often related to disability (e.g., higher EDSS) and disease progression, which lead to higher rates of unemployment, DALYs, and costs. Our results support the early use of NAT (as first-line or early escalation) to reduce the future economic burden of MS. The choice and timing of treatment remain subject of extensive discussion. Currently, an individualized approach is considered, considering various disease and demographic aspects, adverse reaction profiles, as well as local guidelines for decision-making [57].

With broader pharmaceutical competition, lower costs for NAT are anticipated, which could further facilitate its accessibility and affordability. The novel introduction of biosimilars in MS has the potential to significantly reduce DMT acquisitions expenses without compromising therapeutic efficacy [58]. Recently, a phase III trial has demonstrated the similarity between the biosimilar NAT, the first FDA- and EMA approved biosimilar in MS, and its reference medicine [17]. In other medical fields, such as in rheumatology, the introduction of biosimilars likeinfliximab or etanercept has markedly increased the early use of biologic therapies y [59]. This shift has led to significant reductions in healthcare costs while maintaining comparable efficacy and safety. These developments highlight the potential for biosimilars to transform the MS treatment landscape, making high-efficacy therapies more accessible [10].

Moreover, changes in the mode of administration, such as the introduction of subcutaneous formulations and extended interval dosing, may offer additional economic and practical advantages [60, 61]. These approaches can facilitate home administration, reducing the need for frequent clinic visits and lowering associated healthcare costs. When combined with biosimilar therapies, they could further decrease both drug acquisition and administration costs.

Regarding the methodology and results of our study, certain limitations should be considered. As common for simulation-based economic evaluation studies, we employed a model based on combining data from a variety of data sources (Supplementary Tables S2–S9). Although both the input data sources and the model structure were largely consistent with those used in previous work and empirical recommendations, this may have introduced an inherent distortion due to the variability in study settings and patient characteristics across data and populations. To address this limitation with regard to DMT-specific efficacy input data (ARR, 6-CDP), a comprehensive network meta-analysis was incorporated into the modeling process. However, heterogeneity in underlying patient populations, operationalizations, and measurements of endpoints, may still affect the comparison of treatment scenarios under study and limit the generalizability of the findings to some extent. Therefore, while model-based analyses are valuable, the use of large real-world data remains crucial for reliable and generalizable results [62]. The data on the natural history of MS in our model were based on a large historical Canadian dataset (British Columbia data). However, although the British Columbia dataset has a larger cohort size and is more recent than other commonly used data (London Ontario data set), it may not validly reflect the current standard of care and outcomes. In addition, regional differences to the German context may not have been adequately captured.

Additionally, our study was constrained to a 10-year timeframe due to technical limitations of Markov models and the scarcity of long-term (e.g., lifetime) high-quality MS data. MS-specific hazards may change over longer periods of time (e.g., with age and time since diagnosis), which may limit identifying longer term economic and clinical outcomes that might substantially alter the economic landscape [63]. As MS is a chronic disease, this limited horizon may underestimate the true cumulative consequences of DMTs over a lifetime. In particular, direct non-medical costs (informal care) and productivity losses may potentially outweigh the direct DMT costs in a longer term analysis, since they tend to increase more quickly than direct medical cost components. This makes them important factors in understanding the economic burden of MS, which is particularly important in highly active MS, given the close relationship between costs, quality of life, and disability degree [2, 28, 34, 52]. Another inherent limitation of our Markov model is its 'memoryless' property, meaning that it only considers the current health state and does not account for prior disease history or previous treatments when predicting future transitions [64]. While this simplification is common in economic evaluations, it may not fully capture the complexity of MS disease progression, where factors, such as past relapses, disability progression, and treatment responses, can significantly influence future outcomes.

## Conclusion

Our results support the benefits of early use of NAT in people with highly active MS. A 10-year Markov model suggested that early and continuous treatment with NAT significantly reduces disability progression and enhances productivity compared to baseline treatments and delayed therapy initiation. Despite higher DMT costs associated with NAT, the long-term analysis suggests that these are partially compensated by reduced disease management costs. While inherent limitations from the simulation nature of the Markov model are observed, further research should aim to refine these models and explore the impact of NAT and other DMTs in real-world settings.

## Supplementary Information

Below is the link to the electronic supplementary material.

Supplementary file1 (DOCX 147 KB)

## Data Availability

The datasets generated and analyzed during the current study are available from the corresponding author upon reasonable request.
