# Supplementary material for: Cost–consequence analysis of early vs. delayed natalizumab use in highly active relapsing–remitting multiple sclerosis: a simulation study
Source: J Neurol. 2025 Jan 17;272(2):153. doi: 10.1007/s00415-024-12723-4 (PMC11742466; doi:10.1007/s00415-024-12723-4)
Supplement: Supplementary file 1 — Supplementary file1 (DOCX 147 KB) [file 415_2024_12723_MOESM1_ESM.docx]

**Supplementary Material**

**Cost-Consequence Analysis of Early vs. Delayed Natalizumab Use in Highly Active Relapsing-Remitting Multiple Sclerosis: A Simulation Study**

Hernan Inojosa^1,†^, Dirk Schriefer^1,†^, Nils-Henning Ness^2^, Anja Dillenseger^1^, Katja Akgün^1^ and Tjalf Ziemssen^1^

^1^Center of Clinical Neuroscience, Department of Neurology, University Hospital Carl Gustav Carus, Technische Universität Dresden, Dresden, Germany

^2^Hexal AG, Holzkirchen, Germany.

†Contributed equally as first authors.

**Figure S1 - Markov Model Structure**


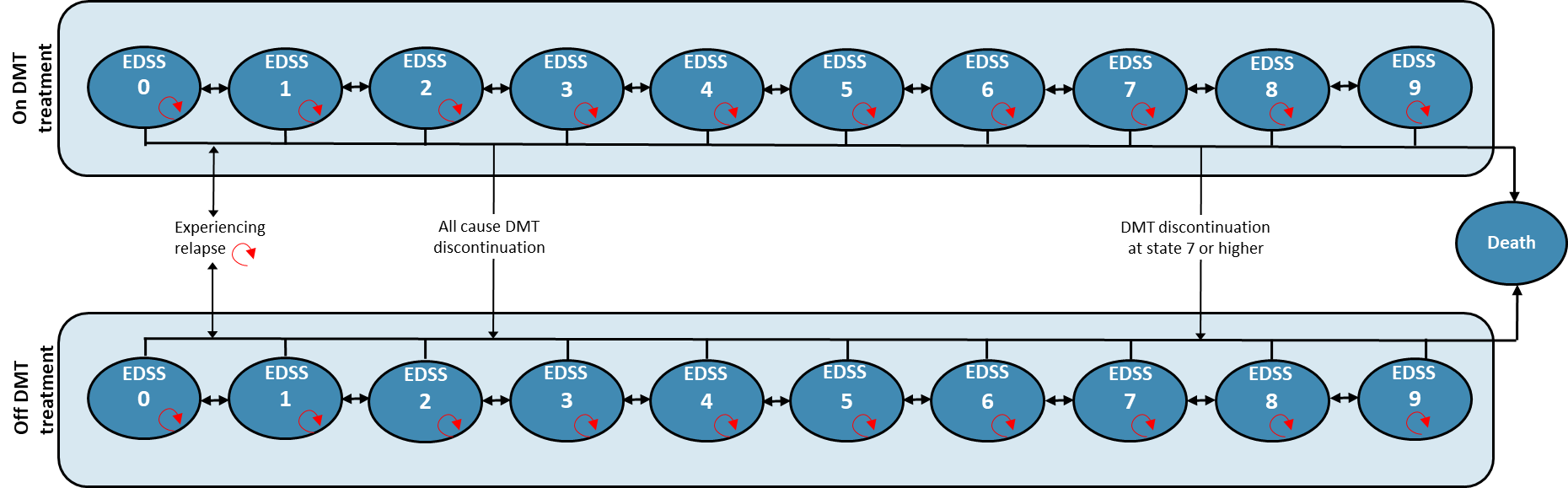


Note: Schematic representation of the Markov model. The model consists of 10 health states based on integer EDSS values: EDSS 0–9 for both a treatment-adjusted (on-DMT) model and a natural history (off-DMT) model, and death (EDSS 10 or natural causes). Over an overall time horizon of 10 years, patients transition between states during one-year cycles. The models starts with a baseline distribution among the health states according to Supplement Table S2. In the figure, health states are represented by circles, and the arrows indicate the transitions between them. A change of more than one EDSS level in a single cycle is allowed. From any state, patients may experience relapse or die. Treatment discontinuation occurs for patients reaching EDSS threshold of 7, or due to other causes. After discontinuation of the initial DMT, patients transition to best supportive care/natural history. Although the EDSS allows for 0.5-point increments, the health states are defined in this model in 1-point increments, which is a common standard in MS-related Markov models. No distinction is made between relapsing-remitting multiple sclerosis and secondary-progressive multiple sclerosis, as diagnosis is frequently done on a retrospective basis after an unclear transition phase.

Abbreviations: DMT = Disease Modifying Therapies, EDSS = Expanded Disability Status Scale

**Table S1 - Key Model Assumptions**

| The patient is always situated within one of the Markov health states. |
| --- |
| The EDSS-wise transition probabilities and DMT treatment effects were assumed to remain constant over time. |
| Patients who discontinued initial treatment followed the natural history progression of the disease and continued on best supportive care. It was assumed that patients were fully adherent prior to discontinuation of therapy. |
| Patients receiving DMTs were assumed to discontinue treatment when their EDSS score reached 7 or higher. |
| No distinction is made between RRMS and SPMS in terms of clinical, economic and mortality data. Consequently, RRMS patients who could theoretically have progressed to secondary progressive MS remain on treatment (unless the EDSS is greater than 7). |
| It was assumed that the retirement age is 67 years and that there are 260 working days per annum in Germany. |
| DMTs exert an indirect effect on mortality by postponing the transition to higher EDSS stages, where the risk of mortality is elevated. It is generally assumed that the administration of DMTs is associated with a reduction in the frequency of relapses and a slowing of the progression of disability, following efficacy input data for the treatment-adjusted (on DMT) model (Table S5). |
| Clinical input data for biosimilar natalizumab assumed to be the same as natalizumab. |

Abbreviations: DMT = Disease Modifying Therapies, EDSS = Expanded Disability Status Scale, RRMS = Relapsing Remitting Multiple Sclerosis, SPMS = Secondary Progressive Multiple Sclerosis.

**Table S2: Model Inputs: Baseline Age, Sex, and Extended Disability Status Scale Distribution**

| Baseline patient characteristics [1] | |
| --- | --- |
| Age (years; mean, SD) | 36.0 ±8.3 |
| Sex (% females) | 70.0% |
| EDSS (%) |  |
| 0 | 5.2% |
| 1 | 29.0% |
| 2 | 33.0% |
| 3 | 20.5% |
| 4 | 9.3% |
| 5 | 2.5% |
| 6 | 0.4% |
| 7 | 0.0% |
| 8 | 0.0% |
| 9 | 0.0% |
| 10 | 0.0% |
| EDSS (mean, SD) | 2.3 ±1.2 |
| EDSS (range) | 0-6 |

Abbreviations: EDSS = Expanded Disability Status Scale

Source: Natalizumab Safety and Efficacy in Relapsing Remitting Multiple Sclerosis (AFFIRM) study (Polman et al., 2006 [1])

**Table S3 - Model Inputs: Probability Matrix of Moving between EDSS States**

| Health state transition probabilities [2] | | | | | | | | | | | |
| --- | --- | --- | --- | --- | --- | --- | --- | --- | --- | --- | --- |
|  | | Transition to EDSS | | | | | | | | | |
|  |  | 0 | 1 | 2 | 3 | 4 | 5 | 6 | 7 | 8 | 9 |
| Transition from EDSS | 0 | 0.695 | 0.203 | 0.073 | 0.022 | 0.004 | 0.001 | 0.002 | 0.000 | 0.000 | 0.000 |
|  | 1 | 0.059 | 0.695 | 0.158 | 0.061 | 0.016 | 0.005 | 0.006 | 0.000 | 0.000 | 0.000 |
|  | 2 | 0.015 | 0.121 | 0.608 | 0.168 | 0.045 | 0.018 | 0.022 | 0.002 | 0.001 | 0.000 |
|  | 3 | 0.007 | 0.050 | 0.120 | 0.544 | 0.091 | 0.058 | 0.116 | 0.010 | 0.004 | 0.000 |
|  | 4 | 0.001 | 0.022 | 0.067 | 0.115 | 0.489 | 0.104 | 0.168 | 0.026 | 0.007 | 0.001 |
|  | 5 | 0.001 | 0.005 | 0.029 | 0.059 | 0.087 | 0.487 | 0.273 | 0.039 | 0.019 | 0.001 |
|  | 6 | 0.000 | 0.001 | 0.004 | 0.025 | 0.031 | 0.041 | 0.741 | 0.109 | 0.044 | 0.004 |
|  | 7 | 0.000 | 0.000 | 0.001 | 0.002 | 0.007 | 0.004 | 0.117 | 0.693 | 0.161 | 0.015 |
|  | 8 | 0.000 | 0.000 | 0.000 | 0.000 | 0.001 | 0.001 | 0.019 | 0.056 | 0.903 | 0.020 |
|  | 9 | 0.000 | 0.000 | 0.000 | 0.000 | 0.000 | 0.000 | 0.002 | 0.006 | 0.174 | 0.818 |

Note: Natural history annual disability progression rates for RRMS. The entries in both matrices reflect the probabilities of moving between pairs of EDSS scores during a single (one-year) cycle.

Abbreviations: EDSS = Expanded Disability Status Scale, RRMS = Relapsing Remitting Multiple Sclerosis

Source: Natural history data from the British Columbia Database (Palace et al., 2014 [2])

**Table S4 - Model Inputs: Relapse Rates**

|  | Natural history annualized relapse rates [3,4,5] | | | | | | | | | |
| --- | --- | --- | --- | --- | --- | --- | --- | --- | --- | --- |
|  | EDSS | | | | | | | | | |
|  | 0 | 1 | 2 | 3 | 4 | 5 | 6 | 7 | 8 | 9 |
| ARR | 0.709 | 0.729 | 0.676 | 0.720 | 0.705 | 0.591 | 0.490 | 0.470 | 0.508 | 0.520 |

Note: Natural history annualized relapse rates for RRMS

Abbreviations: EDSS = Expanded Disability Status Scale, RRMS = Relapsing Remitting Multiple Sclerosis.

Source: The natural history relapse data was sourced from Orme et al. (2007) [3] and Patzold & Pocklington (1982) [4] and used for EDSS-wise calculations of relapse rates [5].

**Table S5 - Model Inputs: Efficacy of Disease Modifying Therapies**

| Treatment effect data [6,7, 8] | | | |
| --- | --- | --- | --- |
|  | Six-month confirmed disability progression (hazard ratio) | Annualized relapse rate | Annual discontinuation probability |
| Glatiramer Acetate | 0.72 | 0.63 | 9.52% |
| Teriflunomide | 0.79 | 0.67 | 11.18% |
| Dimethyl Fumarate | 0.71 | 0.51 | 9.52% |
| Fingolimod | 0.67 | 0.45 | 8.39% |
| Natalizumab | 0.46 | 0.31 | 8.73% |

Note: Input data for the treatment-adjusted model. Estimates were obtained through a systematic literature search employing a network meta-analysis approach (unpublished) [6], in accordance with previously published work [7, 8]. Efficacy data for biosimilar natalizumab were assumed to be the same as those for natalizumab.

Source: Network-Meta-Analysis (Data on file) [6]

**Table S6 - Model Inputs: Mortality Data and Disability Weights**

|  | MS-specific [9,10,11] | | | | | | | | | | |
| --- | --- | --- | --- | --- | --- | --- | --- | --- | --- | --- | --- |
|  | EDSS | | | | | | | | | | |
|  | 0 | 1 | 2 | 3 | 4 | 5 | 6 | 7 | 8 | 9 | 10 |
| Disability weights | 0.000 | 0.011 | 0.021 | 0.110 | 0.199 | 0.256 | 0.313 | 0.617 | 0.772 | 0.926 | 1.000 |
| Mortality weights | 1.000 | 1.300 | 1.600 | 1.680 | 1.760 | 1.840 | 2.710 | 3.570 | 4.440 | 5.310 | 1.000 |
|  | General population mortality [12] | | | | | | | | | | |
|  | Age- and gender-wise data | | | | | | | | | | |
| Probability of dying |  |  |  |  |  |  |  |  |  |  |  |
| Life expectancy |  |  |  |  |  |  |  |  |  |  |  |

Note: Mortality was based on the general population, with the application of MS-specific mortality multipliers by EDSS. Weighted average of the general population all-cause mortality rate is calculated based upon the female to male ratio of MS patients used in the model.

Abbreviations: EDSS = Expanded Disability Status Scale.

Source: Disability weights by EDSS were sourced from Cho and colleagues (2014) [9]. EDSS-wise mortality multiplier were derived from Pokorski et al. (1997) [10] using linear interpolation [11]. Mortality rates of the general population were taken from the morality tables of the German Federal Statistics office [12]

**Table S7 - Model Inputs: Cost and Employment Data**

|  | Economic input data | | | | | | | | | |
| --- | --- | --- | --- | --- | --- | --- | --- | --- | --- | --- |
|  | EDSS | | | | | | | | | |
|  | 0 | 1 | 2 | 3 | 4 | 5 | 6 | 7 | 8 | 9 |
| Employment data [13, 14] | | | | | | | | | | |
| Employed or self-employed (%) | 0.820 | 0.770 | 0.680 | 0.540 | 0.490 | 0.390 | 0.285 | 0.160 | 0.150 | 0.080 |
| Full-time (%) | 0.379 | 0.379 | 0.379 | 0.379 | 0.331 | 0.331 | 0.331 | 0.321 | 0.321 | 0.321 |
| Early Retirement (%) | 0.010 | 0.010 | 0.010 | 0.010 | 0.028 | 0.028 | 0.028 | 0.045 | 0.045 | 0.045 |
| Cost data (resource use/loss) [13, 14] | | | | | | | | | | |
| Direct medical costs (Direct healthcare costs) | | | | | | | | | | |
| Inpatient stays (€) | 516.1 | 645.2 | 1708.3 | 2455.1 | 2678.9 | 3633.5 | 4993.5 | 6462.0 | 8094.9 | 22329.8 |
| Outpatients stays (€) | 41.1 | 107.3 | 183.8 | 187.3 | 363.1 | 209.0 | 211.3 | 194.1 | 347.1 | 171.3 |
| Consultations (€) | 547.0 | 823.3 | 1384.0 | 1547.3 | 1889.8 | 1956.1 | 2073.7 | 2522.4 | 2328.3 | 3537.6 |
| Examinations (€) | 283.2 | 336.9 | 421.4 | 374.5 | 374.5 | 398.5 | 319.7 | 290.0 | 169.0 | 133.6 |
| Medications (non-DMT) (€) | 173.6 | 239.8 | 445.3 | 871.3 | 1274.4 | 1535.9 | 2057.7 | 2515.6 | 2291.8 | 1992.6 |
| DMT-related costs (€) | see Table S8 | | | | | | | | | |
| Direct non-medical costs (Services & informal Care) | | | | | | | | | | |
| Community services (€) | 5.7 | 42.3 | 299.2 | 357.4 | 446.5 | 874.7 | 1948.1 | 4459.1 | 14495.2 | 20140.8 |
| Investments and pruchases | 147.3 | 90.2 | 252.4 | 584.7 | 888.4 | 1490.2 | 2282.7 | 3903.0 | 4100.6 | 1896.7 |
| Informal care (€) | 84.5 | 390.5 | 954.6 | 1622.6 | 2807.9 | 4829.1 | 7092.3 | 14714.5 | 19728.5 | 27836.0 |
| INDIRECT COSTS (Absence from work) | | | | | | | | | | |
| Short term absence (€) | 135.9 | 669.2 | 1070.0 | 951.2 | 809.6 | 479.6 | 231.8 | 97.1 | 161.0 | 0.0 |
| Long-term absence (€) | 3695.2 | 4561.9 | 10025.9 | 15086.7 | 17982.6 | 20912.7 | 23001.2 | 25736.1 | 24743.8 | 36628.6 |
| Cost data (event-specific) [15] | | | | | | | | | | |
| Relapse management costs (€) | 2887.2 | | | | | | | | | |

Note: Cost per patient per year, per EDSS health state or per relapse event, expressed in 2024 euros (€). Employment data expressed as proportion of patients (%) per year per EDSS health state.

Abbreviations: EDSS = Expanded Disability Status Scale, DMT = Disease Modifying Therapies

Source: Employment and cost data inputs were sourced from a large multinational cost study by Kobelt and colleagues (2017) and results from Germany (Flachenecker et al. 2017) [13, 14]. Relapse costs were derived from a German cost study by Ness and colleagues (Ness et al. 2020) [15]

**Table S8 - Model Inputs: Costs of disease-modifying therapies**

| Drug acquisition costs | | | | | | | |
| --- | --- | --- | --- | --- | --- | --- | --- |
| DMT | Route of administration | Dose | Dosage | Pack size (doses per pack-age) | Cost per package (weighted average) | Annual number of doses | Annual Costs |
| Dimethyl Fumerate | Oral | 240 mg | Twice per day | 168 | 2598.0 € | 730.0 | 11288.7 € |
| Fingolimod | Oral | 0.5 mg | Once per day | 98 | 4618.2 € | 365.0 | 17200.6 € |
| Glatiramer Acetate | Subcutaneous | 40 mg/ mL | Three times per week | 36 | 3237.5 € | 182.5 | 16412.1 € |
| Natalizumab (Biosimilar) | Intravenous | 300 mg | Once every four weeks | 1 | 1998.9 € | 13.0 | 25985.4 € |
| Teriflunomide | Oral | 14 mg | Once per day | 84 | 2487.1 € | 365.0 | 10807.0 € |

Note: Costs in 2024 euros (€).

Annual costs associated with acquiring DMT were based on the German pharmacy price schedules. Medications are available on the German market as both originator products and follow-on products such as generics and biosimilars. To account for price differences between these products, packaging prices were weighted by the number of units sold. Since MS is a chronic condition and medications are taken long-term, only the largest package size available from both originator and follow-on manufacturers was used for the calculation of medication costs. The information regarding the dispensed packages was sourced from the pharmacy billing centers in the Pharmascope database.

Abbreviations: DMT = Disease Modifying Therapies

Source: IQVIA PharmaScope® (sell-out data) and Lauer-Taxe® (German drug price directory) as of July 2024 [16].

**References**

[1] Polman, C. H., O'Connor, P. W., Havrdova, E., Hutchinson, M., Kappos, L., Miller, D. H., ... & Sandrock, A. W. (2006). A randomized, placebo-controlled trial of natalizumab for relapsing multiple sclerosis. *New England Journal of Medicine*, 354(9), 899-910.

[2] Palace, J., Bregenzer, T., Tremlett, H., Oger, J., Zhu, F., Boggild, M., ... & Dobson, C. (2014). UK multiple sclerosis risk-sharing scheme: a new natural history dataset and an improved Markov model. *BMJ open*, 4(1), e004073.

[3] Orme, M., Kerrigan, J., Tyas, D., Russell, N., & Nixon, R. (2007). The effect of disease, functional status, and relapses on the utility of people with multiple sclerosis in the UK. *Value in health*, 10(1), 54-60.

[4] Patzold, U., & Pocklington, P. R. (1982). Course of multiple sclerosis: first results of a prospective study carried out of 102 MS patients from 1976–1980. *Acta Neurologica Scandinavica*, *65*(4), 248-266.

[5] Koeditz, D., Frensch, J., Bierbaum, M., Ness, N. H., Ettle, B., Vudumula, U., ... & Ziemssen, T. (2022). Comparing the long-term clinical and economic impact of ofatumumab versus dimethyl fumarate and glatiramer acetate in patients with relapsing multiple sclerosis: A cost-consequence analysis from a societal perspective in Germany. *Multiple Sclerosis Journal–Experimental, Translational and Clinical*, 8(1), 20552173221085741.

[6] Novartis/Hexal. Efficacy of Natalizumab and Other Disease Modifying Therapies for Relapsing Multiple Sclerosis: A Network Meta-Analysis. Data on file.

[7] Samjoo, I. A., Worthington, E., Drudge, C., Zhao, M., Cameron, C., Häring, D. A., ... & Adlard, N. (2020). Comparison of ofatumumab and other disease-modifying therapies for relapsing multiple sclerosis: a network meta-analysis. *Journal of Comparative Effectiveness Research*, 9(18), 1255-1274.

[8] McCool, R., Wilson, K., Arber, M., Fleetwood, K., Toupin, S., Thom, H., ... & Edwards, S. (2019). Systematic review and network meta-analysis comparing ocrelizumab with other treatments for relapsing multiple sclerosis. *Multiple sclerosis and related disorders*, *29*, 55-61.

[9] Cho, J. Y., Hong, K. S., Kim, H. J., Kim, S. H., Min, J. H., Kim, N. H., ... & Kim, W. (2014). Disability weight for each level of the Expanded Disability Status Scale in multiple sclerosis. *Multiple Sclerosis Journal*, 20(9), 1217-1223.

[10] Pokorski, R. J. (1997). Long-Term Survival Experience Of Patients With Multiple Sclerosis. *Journal of Insurance Medicine*, 29, 100-105.

[11] Mauskopf, J., Fay, M., Iyer, R., Sarda, S., & Livingston, T. (2016). Cost-effectiveness of delayed-release dimethyl fumarate for the treatment of relapsing forms of multiple sclerosis in the United States. *Journal of medical economics*, 19(4), 432-442.

[12] Statistisches Bundesamt (Destatis). (2024) Sterbetafeln 2019/2021: Ergebnisse aus der laufenden Berechnung von Periodensterbetafeln für Deutschland und die Bundesländer. Last retrieved February 14, 2024, from <https://www.destatis.de/DE/Themen/Gesellschaft-Umwelt/Bevoelkerung/Sterbefaelle-Lebenserwartung/Publikationen/_publikationen-innen-periodensterbetafel.html>

[13] Kobelt, G., Thompson, A., Berg, J., Gannedahl, M., Eriksson, J., MSCOI Study Group, & European Multiple Sclerosis Platform. (2017). New insights into the burden and costs of multiple sclerosis in Europe. *Multiple Sclerosis Journal*, *23*(8), 1123-1136.

[14] Flachenecker, P., Kobelt, G., Berg, J., Capsa, D., Gannedahl, M., & European Multiple Sclerosis Platform. (2017). New insights into the burden and costs of multiple sclerosis in Europe: results for Germany. *Multiple Sclerosis Journal*, *23*(2_suppl), 78-90.

[15] Ness, N. H., Schriefer, D., Haase, R., Ettle, B., & Ziemssen, T. (2020). Real-world evidence on the societal economic relapse costs in patients with multiple sclerosis. *Pharmacoeconomics*, *38*, 883-892.

[16] Lauer-Fischer GmbH (2024). Lauer-Taxe® 07/2024. Available at https://www.cgm.com/deu_de/produkte/apotheke/lauer-taxe.html
